# Application of Deep Learning in Plant–Microbiota Association Analysis

**DOI:** 10.3389/fgene.2021.697090

**Published:** 2021-10-08

**Authors:** Zhiyu Deng, Jinming Zhang, Junya Li, Xiujun Zhang

**Affiliations:** ^1^Key Laboratory of Plant Germplasm Enhancement and Specialty Agriculture, Wuhan Botanical Garden, Chinese Academy of Sciences, Wuhan, China; ^2^Center of Economic Botany, Core Botanical Gardens, Chinese Academy of Sciences, Wuhan, China; ^3^University of Chinese Academy of Sciences, Beijing, China; ^4^Department of Infectious Diseases, Ruijin Hospital, Shanghai Jiaotong University School of Medicine, Shanghai, China

**Keywords:** plant microbiome, plant-microbiota association analysis, deep learning, plant phenotype, microbiome data analysis

## Abstract

Unraveling the association between microbiome and plant phenotype can illustrate the effect of microbiome on host and then guide the agriculture management. Adequate identification of species and appropriate choice of models are two challenges in microbiome data analysis. Computational models of microbiome data could help in association analysis between the microbiome and plant host. The deep learning methods have been widely used to learn the microbiome data due to their powerful strength of handling the complex, sparse, noisy, and high-dimensional data. Here, we review the analytic strategies in the microbiome data analysis and describe the applications of deep learning models for plant–microbiome correlation studies. We also introduce the application cases of different models in plant–microbiome correlation analysis and discuss how to adapt the models on the critical steps in data processing. From the aspect of data processing manner, model structure, and operating principle, most deep learning models are suitable for the plant microbiome data analysis. The ability of feature representation and pattern recognition is the advantage of deep learning methods in modeling and interpretation for association analysis. Based on published computational experiments, the convolutional neural network and graph neural networks could be recommended for plant microbiome analysis.

## Introduction

The plant-associated microbiota refers to the whole microorganisms colonizing inside the plant organs and on the plant surface, which includes rhizosphere, phyllosphere, and endophyte microbiome (Muller et al., 2016). Numerous species of bacteria, eukaryotes, archaea, and virus inhabit the plant root together. Most of them use the plant carbon nutrient and play a part in plant growth and health (Duran et al., 2018). While under attack of pathogens or insects, plants produce the root exudates that can draw protective microorganisms to resist the invader. When the plant faces the stress, microbiota communities help to strengthen plant tolerance (Yang et al., 2009; Xiong et al., 2020). In return, plant genetics and metabolism can also shape and affect the communities (Li et al., 2018). With the understanding of the associations between microbiome and plants, we can leverage the information of microbiome and predict the host phenotype in advance. Accordingly, we can use microbiological engineering to promote the augmentation of plant production and resistance ability under biotic or abiotic stress (Toju et al., 2018a).

Next-generation sequencing technologies facilitate the collection of genetic information from plant microbiome. Among the sequencing techniques, the 16S amplicon sequencing (Ward et al., 1990) and the metagenomics shotgun sequencing (Sharpton, 2014) are commonly used in plant microbiome studies. The amplicon sequencing focuses on identifying their species and assigning them into certain nodes in the phylogeny tree. However, insufficient reference genome and taxonomy database restricts the taxonomy identification resolution in data analysis, especially in the level of species or strains (Johnson et al., 2019). Comparatively, metagenomic shotgun sequencing makes the acquisition of functional information possible (Breitwieser et al., 2019). The combination of these two strategies can generate comprehensive information (Bulgarelli et al., 2015; Zhang et al., 2016). By means of identification of microbial composition, comparison of different communities, inference of microbial functions, and the metagenome-wide association studies (MWAS) can dig out the associations between communities and plant phenotypes (Wang and Jia, 2016).

Considering the complexity of microbiome data, more powerful and efficient tools are supposed to be explored to interpret microbiome data and find microbiota–plant mutually beneficial relationships (Bulgarelli et al., 2013). In response to the technical demand, machine learning–based methods such as random forest (RF) have been applied to study the impact of the microbiome on plant growth (Chang et al., 2017). Deep learning–based methods such as MetaPheno have been developed for microbiome data pattern learning and data processing in plant–microbiome association studies (LaPierre et al., 2019). The MWAS aim to detect plant phenotype–associated core microbes by plant–microbiome correlation analysis. To identify significant associated microbes, the *p*-values of associations are first estimated by Wilcoxon rank-sum test and then computed by multiple testing adjustments (Xu et al., 2019).

In this narrative review, we talk about the present researches of associations between the microbiome and plant productivity or resistance to stress. We summarize the progress of deep learning (DL) methods and its advantages compared with the classical machine learning (ML) methods and the pipelines for extracting the microbiome trait and perceiving its link to important plant agricultural phenotypes.

## Decoding the Plant–Microbiome Association

### Host-Specific Composition and Beneficial Function

Different from axenic organism, plant grows accompanied by countless microbes in the whole life cycle. The wild microbes contest for nutrients and ecological niches (Dumbrell et al., 2010; Freilich et al., 2011). The winners colonize and form a mutually beneficial symbiont with host plant (Uroz et al., 2019). Serving as the license for microbes to collaborate with plant, molecular basis like genetic determinants and metabolic communications are gradually discovered (Glick, 2014). Consequently, the exploration of association study has evolved into two main steps. First, the composition structure of plant-associated microbial community should be profiled to define the core microbiome taxa, which leads the community assembly and presents distinct features (Agler et al., 2016). Second, the functional evidences indicating causalities or interactions in plant–microbe network under complex and varied environmental conditions would be found (Toju et al., 2018b).

### Methods for Microbiome Data Analysis

The methods for environmental microbiome data analysis are similar with human microbiome study. The MWAS are introduced to establish associations between the microbes and the host genotypes by statistical quantitative comparisons between different samples (Wang and Jia, 2016). The critical step of MWAS is handling the sequencing data with multiple samples. The raw sequence reads are preprocessed by removing sequence errors to control the data quality (Caporaso et al., 2010). The groups of same species are clustered to get operational taxonomic units (OTUs) based on the similarity of sequences of marker genes (Olson et al., 2019). As representative sequences, these OTUs can be used to build the tree for identifying the species based on the sequence's phylogenetic distance and homology database. In this step, the microbial community characteristics like diversity, composition, and host specificity can be found (Bulgarelli et al., 2012; Lundberg et al., 2012), for example, stable and beneficial core microbiome relatives (Schlaeppi et al., 2014), the heritable microbiome taxa in distinct maize lines (Walters et al., 2018), and different microbial taxa and abundance between the different genotypes and rootstock growth stage of the same grapevine species (Berlanas et al., 2019). All of these cases have shown the specific community structure and quantitative characteristic of the plant-associated microbes.

Another vital step is to understand the effect of the microbiota on plants based on functional gene discovery and annotation. The workflow of metagenomics data analysis involves three sub-steps compared with amplicon data: (1) assembling the reads to at least contig level (Sangwan et al., 2016); (2) binning reads, contigs, or genes to bins, species- or strain-level taxonomic units, which initially reduce computational burden for latter analysis (Alneberg et al., 2014); (3) mapping reads or contigs to reference genome, marker genes, annotated contigs or genes, proteins, or metabolic pathways (Quince et al., 2017). The diversity of species and function endows microbial community with resilience and redundancy, which can mediate the states of communities in fluctuant environmental conditions (Hu et al., 2016; Garcia-Garcia et al., 2019). Many microbial beneficial functions come down to the promotion of symbiont health and the resistance to stress (Lemanceau et al., 2017).

The strategies of functional association analysis in recent 10 years are listed in Table 1. The table describes four aspects of microbiome's impacts on host plant and the strategies to explore the function of microbiome from amplicon and metagenome sequence data. The table also presents how to discover the core functional divers and how to establish extensive association between microbiome function and host plant. The researches have tended to directly isolate the beneficial bacteria that were discovered in healthy plant and proved to inhibit pathogen growth or exhibit the plant growth–promoting ability (Haney et al., 2015; Wei et al., 2015). The inoculation experiments have been conducted to test whether participation of selected microbiome consortia has positive effects on host plant under natural suppression from pathogen toxicity, drought, and heavy metal contamination. These studies have provided experimental proof of microbiome influence on host plant. With the development of metagenomic data analysis, the deconstruction of microbiome composition became easier (Hartman et al., 2017). Three issues are focused by the researches, i.e., the discovery of beneficial core microbe taxa, the description of the structure of microbe communities, and the identification of the functional elements (Liu et al., 2020). These effects are embodied in both microbiome community's variations and host plant physical change signs (Luo et al., 2017; Santos-Medellin et al., 2017), plant growth status assessment (Anderson and Habiger, 2012; Purahong et al., 2018), and plant defensive response–related metabolism observation (Rolli et al., 2015; Hou et al., 2018). To seek functional elements including the coding genes (Horton et al., 2014; Finkel et al., 2016), transcripts (Vogel et al., 2016; Zhang et al., 2017), and enzymes (Yasmin et al., 2016), the genes are identified by database homology searches or *de novo* prediction and then attributed into protein families or metabolic pathway. Furthermore, cooperative or competitive associations in microbial community members also have an influence on host plant (Jakuschkin et al., 2016; Blaustein et al., 2017). As a commonly used method, network analysis can help in illuminating the association between microbiome function and plant phenotype. A network presents the correlation of functional elements or pathways in microbial communities. The analysis of functional profiles could be done using MetagenoNets (Nagpal et al., 2020). In addition, the microbial community reconstruction is an effective mean that can validate the linkage between the plant microbiome trait and plant phenotype (Mavromatis et al., 2007; Edwards et al., 2015). The efficiency of these analyses is supported by a powerful bioinformatics tool, which can be found in the MicrobiomeAnalyst platform (Chong et al., 2020; Liu et al., 2020).

**Table 1:** The methods and strategies of functional association study in plant-microbiome.

**Function type**	**Relationship**	**Study and sequencing strategy**	**Approach to define the core plant-associated microbiota and reflect the function**	**References**
**Help plant resist against pathogens**				
Soil-borne fungal pathogens	Disease-suppressive microbes protect plant against pathogen infection	1- Identify key bacterial taxa and genes involved in suppression 2- Isolate key taxa to detect the biosynthetic genes and pathways underlying pathogen control	1- Bacterial taxa with abundance correlated with different levels of pathogen control 2- Gene participating in protection and test by mutant experiment	Mendes et al., 2011
Soil-borne bacterium	The resource competition among resident community and pathogen that impacts on the plant resistance to pathogens	1- Isolate five non-virulent species to do replicated invasion experiments 2- Use interaction network to describe rhizosphere community and response to invader and observe the effect of pathogen invasion	1- Characterize bacterial resource competition networks after inoculating pathogen or 2- Choose core taxa to investigate biodiversity ecosystem function 3- Observe the relationship between the microbial diversity and invasion resistance by path analysis (model) or correlation regression	Wei et al., 2015; Hu et al., 2016
Leaf pathogen(*Arabidopsis thaliana*)	Plant leaf response to colonization by phyllosphere microbiome and pathogen	1- Plant inoculation experiment 2- Conduct RNA sequencing and plant mutant experiment or 3- Directly observe plant physiological change and promotion of plant growth after inoculation	1- Select critical and known certain pathogen species and beneficial bacteria for certain disease 2- Perform differential expression analysis to discover the regulated gene in response to bacterial colonization or 3- Directly detect the activity of defense-related enzymes to reflect the beneficial bacteria function	Vogel et al., 2016; Yasmin et al., 2016
Leaf pathogen (*Quercus robur* L.)	The intra- and inter-relationships in pathobiome community	1- Use ITS1 and 16S sequences to define the taxonomic composition of microbial community 2- Plant inoculation experiment 3- Compare the infection level and select the highly pathogen-susceptible tree to discover relationship between the level of infection with composition of microbial community (use PCA to detect difference)	1- Focus on 13 fungal and 13 bacterial OTUs highly interacted with pathogen 2- Ecological networks to analyze association among species and to distinguish the positive or negative association 3- Network inference to decipher interactions among pathogen with other community members	Jakuschkin et al., 2016
Citrus disease, HuangLongBing	Disease impairs the root microbiome enrichment; core microbiota maintain the stable association between plants under infection of HLB	1- Combine metagenomic and metatranscriptomics approaches to identify the taxonomic and functional properties of root microbiome 2- Perform pairwise comparison of community in different niche and health status/disease symptom severity 3- Use assembled and taxonomic annotated reads to predict unigenes and conduct the functional annotation by blast to KEGG	1- Define the most abundant and dominant bacterial family during HLB disease progression as core bacteria or confirm several main previously proved plant disease–associated bacteria 2- Perform network analysis to identify the mutual relationship between pathogen and core members in community 3- Predict unigenes and pathways by metatranscriptomics	Blaustein et al., 2017; Zhang et al., 2017
Kiwifruit disease caused by Psa	Leaf epiphytic bacteria influence the initial infection process of pathogen Psa pathogen also affects the phyllosphere microbiome	1- Reveal the species- and organ-specific of leaf epiphytic bacterial by 16S (V3, V4) sequencing and classified OTU analysis 2- Observe the change of community structure and biodiversity under pathogen infection 3- Measure three biodiversity indices to find differences in microbiota structures 4- Use multiple regression test (statistical analysis) on microbiome–pathogen infection association	1- Select main beneficial bacteria together with pathogenic consortium 2- Statistically analyze their interactions and correlations, and connect them to the healthy status and plant genotype to study the role of bacteria in promoting plant health	Purahong et al., 2018
**Enhance plant physical tolerance to stress**				
Drought (grapevine)	Some strains have plant-promoting (PGP) traits in drought conditions	1- Select some culturable bacteria 2- Conduct isolation and inoculation experiment to demonstrate the PGP ability 3- Control grow condition to find inducement of ability	1- Find core beneficial microbiome according to previous research 2- Measure the root biomass and metabolite to test the promotion ability	Rolli et al., 2015
Drought (rice)	Drought stress result in root-associated microbiome restructuring	1- Sequencing the 16S (V4 region) to survey the diversity 2- Control the drought treatment to observe the microbiome change in 4 distinct genotypes and 3 field conditions 3- Assess the abundance of OTUs under water deprivation to find core OTUs driving the composition change	1- Perform phylum-level analysis to study the abundance variation, enriched or depleted, in drought-stressed communities	Santos-Medellin et al., 2017
Harsh habitat in desert (salt-secreting desert tree)	Leaf bacteria help plants to adapt to high salinity, high alkalinity, high UV radiation, and periodic desiccation	1- Use 16S amplicon classification to reveal the relationship of microbial community diversity and plant species, environment 2- Assemble the metagenomic reads to know functional characteristics of bacteria exposed to multiple stress factors	1- Binning the contigs into 17 bacterial genomes to locate the core taxa 2- Compare the genomic bins to the closest relatives to reveal function by KEGG annotation, find the main signature: light-sensing genes	Finkel et al., 2016
Cd/Zn contamination in *Sedum alfredii*	Different structure and function of root-associated microbiomes in un- and hyperaccumulating plant genotype	1- Compare two plant genotype root-associated microbiota by 16S profiling 2- Observe the visible symptoms of metal toxicity and plant biomass 3- Predict the function gene related to membrane transporters and energy metabolism to uptake and accumulation of heavy metal	1- Cluster OTUs and taxonomic assign against Greengenes database to identify several genera whose abundance variation can change the metal hyperaccumulation 2- Map 16S rRNA genes to closed reference metagenome profiling and conduct KEGG pathway annotation (use PIRUSt)	Luo et al., 2017
Cd contamination in rice	Different response to Cd-contaminated soil suppression in genotype-specific bacterial community	1- Compare the two rice cultivars—highly or weakly accumulating Cd 2- Analyze the genotype specificity of bacterial community diversity and abundance by bacterial 16S (V3, V4 region) sequencing 3- identify the bacteria taxa specifically enriched in two cultivars, find the rhizosphere microbiome function difference	1- Identify the bacterial taxa specifically enriched in two different cultivars by comparison 2- Find core function taxa related to metal accumulation and activation, and plant growth promotion related to metabolism	Hou et al., 2018
**Promote growth and augment productivity**				
Wheat	Rhizobacteria community that has stable composition and balanced abundance ratio impact productivity	1- Find 16S OTUs that associated with the biomass by regression analysis 2- Define the positive and negative associated taxa and positive-to-negative OTU ratios 3- Observe the relationship between community composition trait with biomass change	1- Select 8 representative OTUs that show the most positive or negative association with biomass 2- The function are reflected by the contribution of bacteria taxa to the biomass measure from statistical aspect	Anderson and Habiger, 2012
*Arabidopsis thaliana*	Genotypic variations in host plant influence and select the microbiome	1- Test hundreds of *Arabidopsis* accessions to determine the effect of level change of WCS365 2- Perform correlation statistical analysis to know which determines the strain specificity of wCS365 among bacteria genotype or host genotype	1- Focus on the proved beneficial bacteria *P. fluorescens* WCS365 2- Test the function of WCS365 by observation of the rhizosphere community changes and plant growth and resistance after inoculating in field experiment	Haney et al., 2015
Soybean crop in field	Different soil microbiome results in high and low productivity fields	1- Shotgun metagenomic analysis to investigate the composition of soil microbiome 2- Use metagenome-wide association studies to determine if abiotic or biotic factors and which taxa associate with high and low crop productivity	1- Different community composition reflect different influence on productivity by Random Forest prediction model	Chang et al., 2017
**Ecological function**				
*Arabidopsis thaliana* leaf microbiome	Host-genetic factors play role in leaf microbial colonization and community structure formation	1- Decipher the community composition by 16S OTUs and ITS2 sequences analysis 2- Look for evidence that host genotypes shape the microbial community by correlation analysis 3- Conduct GWAS and genome-wide SNP detection to reveal influence of host plant loci and gene variance that cause difference in community composition	1- Find most heavily sequenced bacterial OTUs and show the most frequently observed genomic region 2- Define the gene family enriched to biological processes such as defense mechanism and cell wall integrity	Horton et al., 2014
*Arabidopsis thaliana* symbionts	Hub microbes interact with each other and act as drivers to colonization selected by specific plant genotype	1- Establish the community correlation network topology based on 16S amplicon OTUs 2- Use constrained ordination of factors that affect community assembly to find hub microbe and its contribution 3- Correlation analysis to find the factors that impact on community diversity and variation	1- Discover hub microbes that suppress other microbes and control the abundance of other competitors	Agler et al., 2016
Legume plant microbiome	Detailed characterization of *Trifolium* root microbiome is deciphered	1- Describe the root bacteria microbiome composition character with both culture and non-culture methods 2- Conduct climate chamber experiment to know whether field conditions do matter in differing the composition 3- Perform inoculation experiment to evaluate the plant–microbiota interaction potential by scoring effects of the bacteria on plant growth and thus find what genus generates positive or negative impacts	1- Identify the high relative abundant root microbiome OTUs 2- Discover the function traits by finding out nutrient-providing *Rhizobia* bacteria and protection from pathogenic disease	Hartman et al., 2017
Grass microbiome	Microbiome function diversity and complexity traits can predict nutrient cycling ecosystem function	1- Quantify 10 functions reflecting ecosystem nutrient cycling to asses 3 plant functional groups 2- Profile the soil bacteria and fungi communities by 16S and ITS OTU analysis 3- Construct microbial association networks to analyze the linkage density between OTUs, functional complexity, community traits, and so on	1- Use randomization test to assess whether each taxa predicts particular ecosystem functions 2- Use net regularization to predict all taxa community by feature selection 3- Infer the positive or negative functional contribution of each OTU	Wagg et al., 2019
Soil microbiome	Soil microbiome composition indicates and predicts the soil physico-chemical traits	1- Collect soil physico-chemical variables from 606 sites to characterize the soil environment 2- Sequence the 16S (V3–V4) amplicons to represent the soil microbiome composition 3- Concentrate on the bacteria OTUs after taxonomic annotation by Greengenes database reference 4- Use random forest model to connect OTUs to soil trait values and scores	1- Use bacterial community composition to predict soil conditions by prediction model	Hermans et al., 2020

## Plant Phenotype Prediction: A New Angle

### Predicting Phenotype Based on Plant Microbiome

**Figure 1:**
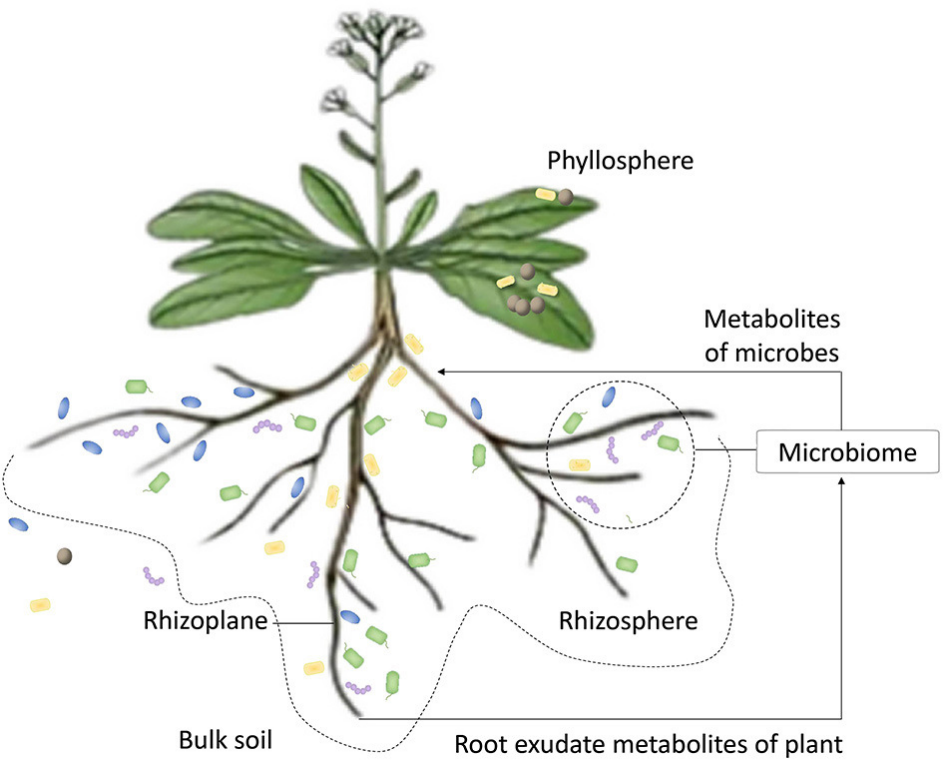
The scheme of plant on symbiosis with microorganisms. The plant provides microbial community carbon, nitrogen nutrient substance, and some root exudate metabolites, such as amino acids, sugars, and organic acids. These substances can drive the enrichment of microbes from bulk soil to the region of rhizosphere. Microbes that are adapted to the exudate metabolites can pass the selection by plant and finish colonization. Microbes also produce metabolites, such as hormones and secondary metabolites, to participate in the regulation of metabolism.

The traditional research patterns of plant-associated microbiome involve profiling the microbiome composition and finding metabolic function units (Figure 1). Recently, some works have turned attention into direct prediction of plant agronomic traits according to the features of overall microbial community. The stable heritability of several certain core microbiome taxa with certain function units can signal some characteristics in terms of plant genotypes (Lundberg et al., 2012), plant adaption to stress (Zhang et al., 2020), and plant productivity (Jin et al., 2017). This allows us to speculate on the quality of host plant according to microbiome data. The resolution limitation of microbe fingerprinting and the knowledge bottleneck of complex microbiome are two challenges in microbiome data analysis. To address these challenges, the tendency of research has transformed the analysis from finding unilateral correlation to forecasting overall phenotype (Chang et al., 2017). First, we cannot absolutely reach species- and strain-level high resolution because of the incompleteness in taxonomic annotation. The subtle differences in strains that may influence the impact of microbiome on host such as the differences in pathogenicity or toxicity of strains (Blaustein et al., 2017), and the species without taxonomic or functional annotation both can leave us a dilemma whether we can ignore them in data processing (Mendes et al., 2011). Second, as shown in Table 1, the interactions among microbiome community members or between microbiome and host are bilateral and interlaced. The interference factors are hard to be excluded by a few sample sets. These complex associations make the prediction of phenotype difficult. In general, more attention is paid to find the unilateral association between microbiome and host. In fact, the dispersive features and multilateral associations are synergistically determined by plant genotypes (Walters et al., 2018), soil conditions (Santos-Medellin et al., 2017), degree and time of stress (Blaustein et al., 2017), and other invisible factors. In other words, it is the whole community that performs the function on host rather than the merely several core taxa (Wagg et al., 2019). Similarly, the host plant phenotype should be considered as the aggregate consequence of many changeful and mutually restricted influence factors from plant-associated microbiome traits and natural environmental variables.

### New Requirement of Data Integration

The understanding of multilateral associations needs adequate information such as the microbes at different taxonomy level, the multi-omics data like metagenome and transcriptome, the characters of core microbiome taxa and whole communities, and the influences of synergistic factors on host (Xiong et al., 2020). These different types of data should be integrated to directly and efficiently predict the plant's important agricultural phenotype with which the plant growth states can be understood (Chang et al., 2017; Gu et al., 2020) and then be managed to improve the adaptation of plant to the environmental change and stress (Hermans et al., 2020). With the ability of representing the metagenomic sequence data and extracting the important features and associations in the host–microbiome system, supervised learning methods for the high-dimensional and complex microbiome data have sprung out. As a branch of machine learning method, the deep learning methods have a flexible model to process high dimension data (Zou et al., 2019). Thus, the deep learning methods provide the advantage of studying the plant–microbiome associations that link comprehensive microbiome genetic information to host phenotypic traits and physiological states of plants (Knights et al., 2011a; LeCun et al., 2015).

### Representation Learning for Microbiome Data

Four kinds of DL models have been introduced to model genomic data (Eraslan et al., 2019). The tutorial of model designs and training steps tailored for different types of data has been provided. The features of genomic sequence data can be represented by k-mer counts (Tu et al., 2014; Liu et al., 2017), position weight matrix (Stormo, 2000; Alipanahi et al., 2015), and network-structured data like protein–protein interactions. There have been some reviews that summarized the applications of ML in human microbiome data analysis for different tasks. The classification of microbial species, the prediction of host phenotypes and ecological environments, the investigation of interactions between community members, and the prediction of associations between microbiome and disease are the key tasks (Qu et al., 2019; Zhou and Gallins, 2019). More and more DL models show advantages in human metagenomics data in detecting biomarkers that characterize the microbiome traits and host phenotype, such as MetaPheno (LaPierre et al., 2019) and MDeep (Wang et al., 2020). The ML method has succeeded in predicting productivity based on plant soil metagenomic data. Therefore, the DL methods can also help to improve performances in plant-associated microbiome data analysis. In this review, we summarize recent DL methods designed for metagenomic data in recent years. We discuss the specific role of DL methods in learning the pattern from microbiome data and modeling for prediction task. We also discuss the advantages of DL methods in dealing with problems in practical limitation of plant microbiome compared with traditional ML methods (Chang et al., 2017).

**Figure 2:**
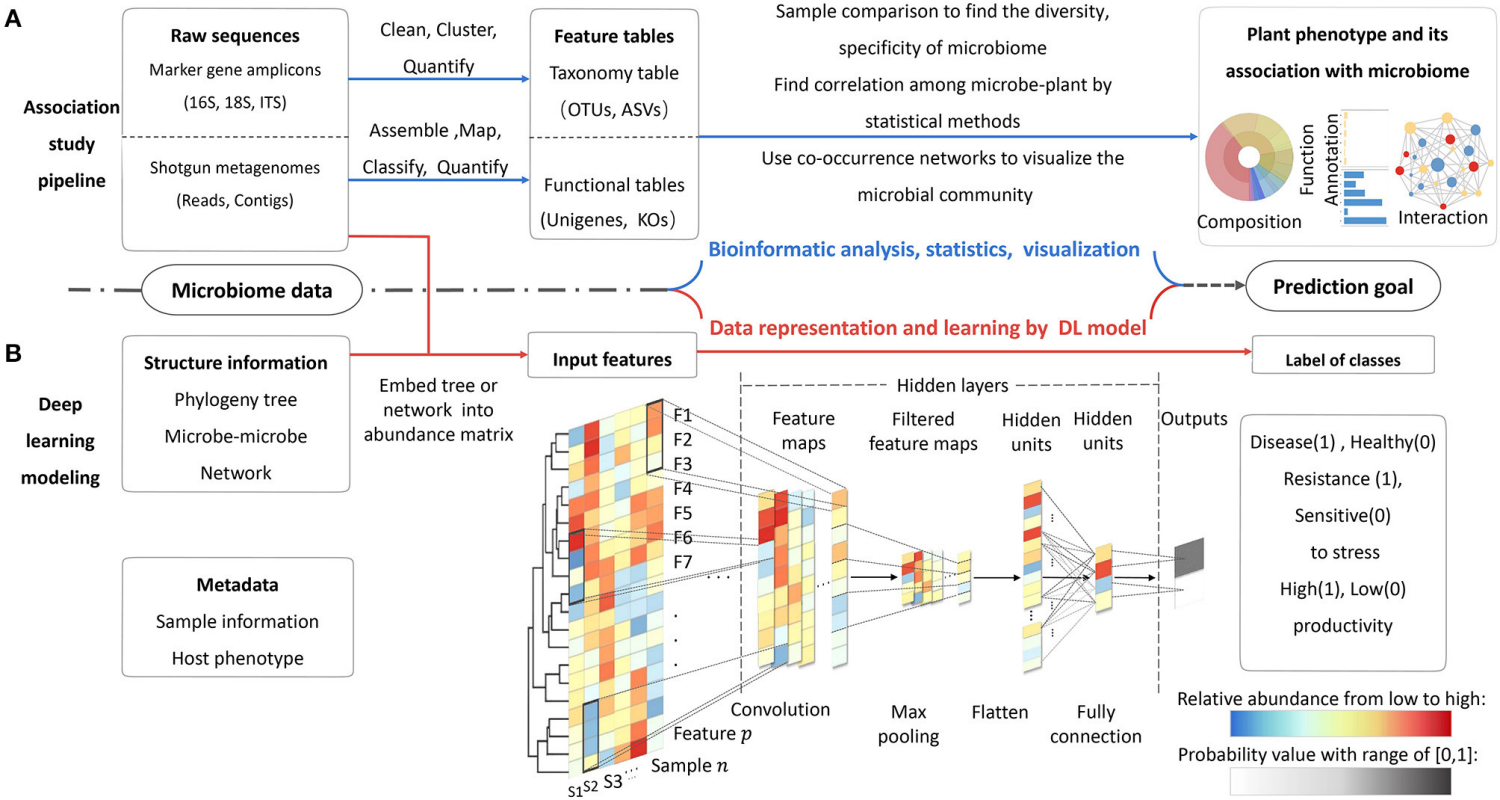
Two routes of microbiome data analysis. **(A)** The processes of traditional association study by bioinformatics tools. **(B)** The processes of microbiome–phenotype connection by DL model. The model can integrate more information of data at the same time. In this example, a binary-classification CNN model, in which the abundance of features matrix is input and the outputs are the probability values of two class labels (0, 1). In hidden layers, operations of convolution and maximum pooling can extract the informative features by auto-updating the weight of each unit (feature). The filters in the black window down-sweep the input feature and get new matrices for each sample. The exact number of layers will be decided according to prediction accuracy about 3–5 layers based on experience. The last two layers are fully connected with their weight. The weights of connection represent the importance values of the units, as shown by the black dotted line with different color depths. Activation function at last layer can produce the probability vector that can be used for the final classification or prediction.

The microbiome data harbor three aspects of information, i.e., the taxonomic and functional compositions of all microbial species, the interactions among each member in community, and the associations between microbiome and host plant in a certain environment (Figure 2A). The objective of modeling is learning inherent patterns and finding significant features from this information. A training model should be able to precisely predict the plant-associated knowledge of interest. In a training study, the implementation follows four steps: (1) prepare dataset for a prediction task; (2) construct suitable model architecture; (3) train and optimize the model; (4) finish end-to-end prediction task and test performances on new datasets. There are also detailed implementations and guidance of model training. The main program of modeling for MWAS includes three steps: (1) prepare and parse the input data; (2) learn the pattern of data and identify the relevant features by DL models; (3) execute the classification or regression task based on causal correlation (Li et al., 2019; Zou et al., 2019).

### Detect Biomarkers From Data

The input data include 16S amplicon or shotgun sequencing data. The sequencing data can be numerically encoded into one-hot matrix (Pan and Shen, 2018) and feature table that are processed by classical bioinformatics analysis pipeline like OTU table (Bolyen et al., 2019) or amplicon sequence variant (ASV) table (Callahan et al., 2016). The first step of MWAS is to discover the microbial biomarkers from high-dimensional, sparse, and noisy metagenomic data. Similar sequences are clustered and aligned to the databases of marker genes, i.e., 16S, 18S, and ITS (internal transcribed spacer). The sequences with the maximum similarity to the reference sequences are the representative sequences, namely, OTUs. The OTUs with high abundance in samples represent the core taxa that reflect component specificity in microbiome community. For the shotgun sequencing data, the predicted function genes act as biomarkers to represent the function signature (Segata et al., 2011).

The limitation of data analysis is that existing methods of feature representation like OTU tables are not comprehensive enough to give a complete picture of the whole microbiome. For instance, a single threshold like 97% for taxonomic classification may not be optimal for all kinds of microbiome datasets. What is more, the core taxa may belong to multiple different taxonomic levels ranging from class, genus, and species. In this case, the selected microbial signatures that fall in different classification levels are much harder to be represented (Knights et al., 2011b). For the ML methods, the feature representation was manually designed by microbiology experts who adopt OTUs, ASVs, or alpha-diversity to describe the data feature composition and abundance. This kind of feature engineering and pattern learning in DL tasks is automatically conducted by the multilayer perception in deep neural network (DNN). The first advantage of DL is capacity of data feature representation compared with ML. Via multiple hidden layer process, the hidden features of input data will be learned and represented layer by layer, making the connection of input signal with output prediction target much tighter (LeCun et al., 2015). As a basic deep neural network, deep feed-forward network is made of input layer, hidden layer, and output layer. Actually, to improve the prediction performances of deep learning, optimizing the representations of data features is a general strategy. Commonly, the ML methods treat tables of OTU abundance as input features that represent the composition and quantity information. This has worked well in human microbiome sequence classification especially in RFs and support vector machines (SVM) (Statnikov et al., 2013). As for metagenomic shotgun sequencing data, MetaphlAn2 can generate taxonomic profiles shaped like OTU tables from metagenomic sequence assigned to marker gene or functional gene database. The input datasets are generally represented by the OTU tables in a tabular format, which show the abundance of each taxa in each sample. Identification of biomarkers is carried out through data representation and feature extraction (Truong et al., 2015).

### Data Representation and Feature Extraction

The OTU features collect and represent a subset of similar sequences, but the DNA mutations are ignored when disturbing cluster division (Koeppel and Wu, 2013). In some models, the k-mer representation has been proved to outperform on the same datasets owing to less computation complexity and independence on reference database (Vervier et al., 2016; Asgari et al., 2018). The k-mer counting means the frequency of all subsequences of different length *k* in a given sequence data. The embedding representation has shown the advantage of encoding the 16S amplicon sequence into low-dimensional space and preserving species-level resolution of sequences. These low-dimensional representation methods could emulate the original features of vast sequence data and preserve the sequence similarity and difference. The k-mers can be encoded to vectors through embedding and then be fed into the input layer (Woloszynek et al., 2019).

In the OTU-feature representations, a cluster of similar sequences are represented by a representative sequence. This method can correct the sequencing errors but erase subtle differences of sequences and result in taxonomic classification bias (Callahan et al., 2017). Short sequences with small *k* cannot detect the subtle differences. The larger *k* tends to cause the curse of dimensionality. To alleviate this problem, sequence can be mapped into low-dimensional space through embedding. The embedding method transforms feature maps into image format by t-SNE (t-Distributed Stochastic Neighbor Embedding) (van der Maaten and Hinton, 2008). A study of 2D embedding has shown how to map species-abundance datasets to 1D or 2D images for convolutional neural network model (Hai Nguyen et al., 2017). The 1D image arranges the features according to increasingly taxonomical order. The colors with different depths are used to distinguish the abundance. The black (white) color means presence (absence) of certain species (Figure 2B). The 2D images are a low-dimensional projection of feature points from all samples by t-SNE (van der Maaten and Hinton, 2008). As a prior knowledge of evolutional relationship in a microbial community, phylogenetic tree helps to understand the inherent structure of microbiome data. This kind of spatial information among numerous features may not be captured by OTUs or k-mers (Albanese et al., 2015; Fioravanti et al., 2018).

After integrating the phylogenetic tree into matrix, the feature map has both quantitative information and the spatial relationship of feature nodes in the tree. The phylogenetic trees are constructed by OTU abundance tables. The nodes in trees are OTUs that ranged based on the evolutionary distance between different representative sequences. Then trees with abundance and location information are embedded into a matrix where the root node is on the top left corner and each row is filled with the child nodes, and vacant position is padded with 0. Multi-dimensional scaling (Cox, 2001) can also preserve the defined tree distances between two OTU points when mapping OTUs into a subspace (Fioravanti et al., 2018). This format of matrix is suitable for convolutional neural network, which is especially good at handing the pattern recognition of 2D image–like input data with pixels. In addition, given the complex inside relationship between microorganisms, a method that built a sparse correlation network from OTU abundance data and then embedded the network into the DL model (GEDFN) has been raised. Embedding feature maps into graph-like matrices and outperforming the tree-based representation and traditional ML feature selection, this model is a reasonable representation (Zhu et al., 2019).

The significance of appropriate numerical encoding and representations for this kind of complex microbiome data in subsequent modeling is self-evident. It enables the better interpretation of complex and structurized microbial data, which helps to fully leverage the information of microbiome data to predict the host phenotype. As underlying signals, different features make the microbiome communities different. So the features should be precisely identified for phenotype prediction (LaPierre et al., 2019). There are three types of features including the taxa abundance, the k-mer distribution of raw reads, and the function gene. The OTU abundance and k-mer distribution are quantitatively statistical features of the sequence itself. The embedding methods can map the high-dimensional features into lower-dimensional plane space or embed the quantitative characteristics into phylogenetic trees. This belongs to image-based and tree-based representations (Hai Nguyen et al., 2017).

After feature representation and dimension reduction, feature selection is another data preprocessing task to improve the accuracy of the prediction model. The goal of feature selection is to select the most relevant feature subset and remove the irrelevant feature and redundant feature. Five kinds of feature selection methods have been conducted (Statnikov et al., 2013). All these methods have improved the classification accuracy. The traditional feature extraction methods (i.e., PCA and PCoA) for OTUs do not improve the performances in prediction and classification due to the information consumption in excessive dimension reduction and multilevel feature filtration. Therefore, the feature engineering needs more simple and effective methods. The ensemble feature selection methods (Pes et al., 2017; Seijo-Pardo et al., 2017) applying to MWAS have been introduced and tested (Zhu et al., 2020). The MDeep model has been developed to simulate the phylogenetic tree structure of microbial taxa at different taxonomical levels. It indicated that convolutional neural network could automatically learn representation and map a complex feature to the simple one by convolutional multilayers (Wang et al., 2020).

The step of feature selection alleviates the data complexity and high dimension. It decides the most discriminative features among samples and identifies the most relevant core biomarkers for microbiome-associated phenotype of host. Instead of classical ML feature selection methods, the DL models handle the raw data better since they can learn the representation and extract important features automatically in an end-to-end manner. Some features that are endowed with high importance score will be reflected on the relatively larger connection weights of neural node during DL prediction model training. This operation can leave out the feature selection steps for reduction of excessive loss of information (Ditzler et al., 2015).

## Deep Learning Models for Prediction

The DL models with high computational efficiencies include convolutional neural networks (CNN), recurrent neural networks (RNN), and graph convolutional neural networks (GCN). Here, we introduce three categories of tasks that the DL methods applied in microbial data analysis (Eraslan et al., 2019).

### Deciphering Species Composition of Microbiome Data

Microbiome data are characterized by a mixture of known and unknown species that makes data high-dimensional and sparse. The application of supervised classification of microbiota has been demonstrated to be feasible (Knights et al., 2011a). An open question has been raised as to how to incorporate the phylogenetic information into OTUs. As PhyloRelief has indicated, the tree-based representation can deal with this problem (Albanese et al., 2015).

The CNN and deep belief network (DBN) architectures have been used for taxonomic classification based on emulated 16S reads generated by artificial simulation tool Grinder (Angly et al., 2012). The classification models have been applied on each taxonomic level from phylum to genus based on k-mer representation (Fiannaca et al., 2018). This model has been proved to outperform the considered baseline-RDP classifier. For instance, with *k* = 5 prepared taxonomy annotation table and k-mer frequency table are transformed into a matrix with k-mers or taxonomy label (such as genera) of each sequence ID as rows and sequence ID as columns. This matrix is the training data *X* for microbial communities. Classes of each datasets are transformed into numerical labels as training *Y* for each sequence ID, such as −1, 0, and 1. In both CNN and DBN, features of input *X* can be mapped to a lower dimensional feature space through the hidden layers. The feature maps are then sent to a fully connected layer to conduct the binary classification. Besides, we can compare the performances of separated models with different *k* to define the best *k* setting. In this way, the models can finish unsupervised feature extraction and supervised classification in architecture, simplifying the feature engineering and supervised learning task in machine learning (Wang et al., 2007).

The Ph-CNN is a CNN architecture endowed with auxiliary information of hierarchical structure by tree-based representation (Fioravanti et al., 2018). It processes data in a format of digital image with pixels. It uses a designed Phylo-Conv keras layer that sums the leaves of the tree in which each leaf represents the abundance of OTU at a certain position on the phylogenetic tree. Then it uses filters to slide over all points of input variables and convolve for detecting neighborhood of leaves. The model ranks the neighborhood of each OTU based on distance, which can help to detect most discrimination taxa among different samples. DeepMicrobes model has developed a toolbox of taxonomic classification for metagenomic sequences with more complete fragments than amplicons (Liang et al., 2019). Based on k-mer representation, the RNN models are trained on reference genomes. They have built models for each taxonomic level of phylum, class, order, family, and genus with different read lengths. The models are built to distinguish similar taxa. The researchers have also compared the k-mers and one-hot encoding methods in other architectures like ResNet-like CNN models (Jaganathan et al., 2019) and LSTM models (Hochreiter and Schmidhuber, 1997). The results have shown that k-mer representation could improve performances of classification by dealing with short sequences.

It is impractical for all real sequenced data to be equipped with reference genome and known taxonomic category especially in the natural environment. The collection of worldwide plant microbiome data sample is far more hard and costly than human gut microbiome, which makes reference genomes and labeled data insufficient. The applications of DL modes are more powerful for mining microbiome data and processing new data independent of reference database than classical ML methods.

### Functional Analysis by Deep Learning Methods

Functional gene prediction is a contributory as significant taxa's marker gene identified in association analysis. However, gene finding in metagenomic sequences is limited by incompleteness and fragmentation. The gene caller has been developed to extract complete and incomplete open reading frames (ORFs) from short reads and recognize coding ORFs by ML classification methods (El Allali and Rose, 2013). An integrative framework has been built to predict the functions of microbial communities by ML method that combined composition structure of microbial communities with knowledge contexts such as phylogenetic tree structure in communities (Wassan et al., 2019). This method aims to discover the biomarkers (OTU features) and assess their functions. A CNN model has also shown the feasibility of DL in functional annotation of genome sequences (Khodabandelou et al., 2020). The authors have recognized the short sequences with certain known functions, such as functions of the promoters in different species. A CNN model (CNN-MGP) has been built to recognize genes from raw metagenomic DNA sequences without manual feature selection (Al-Ajlan and El Allali, 2019). The model can automatically learn the features of sequences within the regions of certain function elements, and distinguish coding and non-coding regions according to ORF recognition. The authors have encoded *L*-length ORFs into one-hot matrix (L^*^4) as numerical representation (Al-Ajlan and El Allali, 2019). They have specially chosen the one-dimensional CNN that is suitable for DNA sequence data. They have built models for each different GC content interval range, and then used input layers and convolution layers to extract features. Lastly, they have used a fully connected layer with non-linear activation function to generate output-probability values. The post-processing step involves a list of some candidate genes with probability values above 0.5. The greedy algorithm are used to iteratively select fragments with maximal probability and remove the overlap smaller than 60 bp (Hoff et al., 2008). The method can be used for function annotation on metagenomic reads in a supervised manner, by reference of gene annotations in GenBank, KEGG Orthologs or Pathways, FunGene (Fish et al., 2013), COG (Galperin et al., 2019), and MG-RAST (Wilke et al., 2015).

### Directly Predicting the Host Phenotype

An important step forward in association study of microbiome and host is the application of ML models in MWAS. In this study, the RF methods are used to perform a binary classification and divide the samples into high or low productivity with a threshold value based on the certain amounts of microbial taxa at each taxonomic level (Chang et al., 2017). The model has calculated the importance of each taxon that contributes to indicate the corresponding sample productivity traits. Then more researchers have begun to explore the DL methods to conduct this end-to-end way of prediction. The Ph-CNN model has used gut microbiome data from patients with six kinds of inflammatory bowel disease (IDB) to divide the data into different classes based on OTU abundances and phylogenetic distance information (Fioravanti et al., 2018). The study of MetaNN has also introduced the classical ML methods (Lo and Marculescu, 2019). The authors have tested DNN and CNN models on differential OTU abundances to divide the disease states of IDB and Type 2 diabetes with data perturbation. The PopPhy-CNN has provided a paradigm of CNN framework linking the metagenomic data with host phenotype (Reiman et al., 2020). The framework includes the metagenomic profile representation, important feature extraction, and disease prediction. They have evaluated DNN methods compared with other ML methods on predicting samples from 16S gene with k-mer based representation, which demonstrates the advantages of DL models at large datasets (Asgari et al., 2018).

In specific research of plant, the collection of a large number of duplicate samples covering all the conditions is unpractical. For instance, we cannot collect the microbiome samples from all possible temperatures, soil pH values, and concentrations of salt or heavy metal. This limitation results in the small size of training dataset that causes the over-fitting of models. Most researchers generate simulated samples to augment data and adopt cross-validation or bootstrapping methods to divide the training and testing dataset. The Deep Forest based on random forest has been adopted in a robust ensemble model with less parameter to tune. It is a good attempt to combine deep cascade structure with ML method for the insufficient large datasets (Zhu et al., 2020).

To be described as variables, the plant-associated microbiome data should contain enough samples and specific phenotypic traits. The available datasets that can be used for training DL model to do prediction task are summarized in Table 2. These datasets were generated by some projects of association studies on crop productivity (Chang et al., 2017; Jin et al., 2017), drought stress (Santos-Medellin et al., 2017), and plant disease (Zhang et al., 2017, 2020). By deciphering the composition structure and functional hits, these studies intended to make the related comparisons for different host phenotypes, for example, the states of drought or control, the states of health or HLB disease, the resistances to disease, and the states of productivity. With the results, we can define the classified unit (OTUs) with its abundance value as the predictive variables and use trait of each sample as the response variables (labels) to conduct the prediction task through DL models. The abundance table is in the shape of matrix *N*^n*p^, where *N* is a set of natural numbers, and *n* and *p* are the number of samples and features, respectively. Calculated by the OTU processing method MetaNN, each vector of samples *d*_*i*_= [*d*_*i*1_*, d*_*i*2_*, d*_*i*3_*,…, d*_*ip*_] represents relative taxonomy abundances of features. These OTUs serve as input features for the neural network models. The classes of sample label can be defined as data points such as 0 (low productivity) and 1 (high productivity) (Lo and Marculescu, 2019).

**Table 2:** Datasets of the plant-associated microbiome.

**Datasets**	**Sample size**	**Number of features (total)**	**Classes**	**Prediction tasks**	**References**
Drought-amplicon(NCBI) (rice)	216+216	1,461 OTUs (genus)	2	Classification of state of drought (1) or watered (0)	Santos-Medellin et al., 2017
HLB disease-metagenome(NCBI) (citrus)	6+6	7,577,213 unigenes (bacterial)	2	Classification of healthy (0) or HLB disease (1) sample	Zhang et al., 2017
Productivity-amplicon(EBI) (foxtail millet)	2,882	16,109 OTUs	30	Prediction of 30 productivity group according to the grain weight per plant (1~30 g)	Jin et al., 2017
Productivity-metagenome(MG-RAST) (soybean)	6+6	7,073 OTUs (genus)	2	Prediction of high (1) or low (0) productivity	Chang et al., 2017
Disease-amplicon and metagenome(NCBI) (cassava)	30+30+30	166,097 OTUs (16S)22,339 OTUs (ITS)	4	Prediction of response level of sensitive (−2), medium sensitive (−1), medium resistance (1), resistance (2)	Zhang et al., 2020

There are multiple factors that affect the host plant phenotypes. For instance, the factors affecting yield include genotypes of host, states of growth, and resistances to stress. The microbial communities also make differences. The previous studies have used single factor and microbiome composition data to model and predict host phenotype of interest. After finding that the rhizoplane microbiome plays a part in sample differentiation, the researches have turned to the rhizoplane microbiome taxa and their correlation with the host phenotype (Zhang et al., 2017). For the large capacity of high-dimensional data, the DL model is used to aggregate the data of different types and batches. There are three types of microbiome data that can be aggregated, i.e., the abundance information of taxa, the tree or network structure information of communities, and functional unigenes.

The key challenge of the plant microbiome data analysis is the insufficiency of sample number in plant association studies, which may cause over-fitting of model. Therefore, we recommend the pre-training model based on the same class data such as environment metagenome reference sequence in MetaMetaDB (Yang and Iwasaki, 2014) and rice-associated microbiome data (Kim and Lee, 2020). Current data are scattered over the different separate study, so the comprehensive benchmark datasets are desperately needed for the plant microbiome. The cross-validation (CV) is an efficient method in model training (Xing et al., 2020). For instance, the *k*-fold CV divides the observation datasets into *k* groups with the same size. Each *k* fold is set aside for validation and the accuracies are calculated in epochs. The whole process produces *k* average MSEs. The results of *k*-fold CV is estimated by averaging these values. By comparing the accuracy of 10-fold cross-validation, the CNN model with 2D kernel improved the accuracy by up to 5.6% (Hai Nguyen et al., 2017). Due to the better ability of pattern learning, the CNN model with tree-based representation performed better than RF and SVM at the species level (Reiman et al., 2020). With 5-fold cross-validation, the graph embedding DNN model GEDFN largely outperformed to the SVM model on the same training set (Zhu et al., 2019). These cross-validation tests have proved the good performance of deep learning models prior to machine learning models. Besides, we can use Grinder (Angly et al., 2012) and CAMISIM (Fritz et al., 2019) to generate simulated metagenome abundance data and append them into training sets. Most OTUs (features) are filtered by common bioinformatics tools in the universal threshold of 97%.

## Conclusion

The association study between plant microbiome and host plant phenotype can be considered as a data mining strategy that extracts composition and quantity features from microbiome sequence data. It facilitates the understanding of plant microbiome traits and their impact on phenotype of host plant. The deep learning models have become predominantly methods on dealing with microbiome data which features by multi-species mixture, high-dimensionality of data, and sparsity due to incomplete annotation knowledge. Until very recently, to conduct a prediction task and statistical association between microbiome data and host phenotype, researchers have used machine learning methods to process. However, the requirements for better integration of more information in microbiome data to predict the agronomic trait of host plant more precisely have presented opportunities for deep learning methods. The advantages include higher capacity for high-dimensional data, flexibility architecture for processing data of different formats, and good ability in representation of intrinsic features and structure in data. This allows the deep learning models to automatically learn complex structural pattern and quantitative characteristics of plant-associated microbiome data. These advantages make deep learning models stand out in prediction tasks. Based on existing study strategies of association discovery, the application of deep learning models is a new angle of building relationship between the microbiome data and the host. The models of pattern recognition such as convolutional neural networks and graph neural networks can assist in some critical steps of association researches. Moreover, more attempts need to be conducted to figure out which deep neural network can best fit the real plant microbiome data and how to adapt transfer learning to use the finite data resources of environment microbiome for processing changeable plant microbiome under a flexible natural environment.

## Author Contributions

XZ and ZD conceived and designed the research and approved the final article. ZD performed the research. ZD, JZ, and JL wrote the original draft. All authors contributed to the article and approved the submitted version.

## Funding

This work was supported by the National Natural Science Foundation of China (32070682), Technology Innovation Zone Project (1816315XJ00100216), and CAS Pioneer Hundred Talents Program.

## Conflict of Interest

The authors declare that the research was conducted in the absence of any commercial or financial relationships that could be construed as a potential conflict of interest.

## Publisher's Note

All claims expressed in this article are solely those of the authors and do not necessarily represent those of their affiliated organizations, or those of the publisher, the editors and the reviewers. Any product that may be evaluated in this article, or claim that may be made by its manufacturer, is not guaranteed or endorsed by the publisher.
